# Identification of Three *POMCa* Genotypes in Largemouth Bass (*Micropterus salmoides*) and Their Differential Physiological Responses to Feed Domestication

**DOI:** 10.3390/ani14243638

**Published:** 2024-12-17

**Authors:** Jie Hu, Jie Yang, Huan Zhong, Qifang Yu, Jun Xiao, Chun Zhang

**Affiliations:** State Key Laboratory of Developmental Biology of Freshwater Fish, Engineering Research Center of Polyploid Fish Reproduction and Breeding of the State Education Ministry, College of Life Sciences, Hunan Normal University, Changsha 410081, China; sunshine990725@163.com (J.Y.); zhonghuanzh@126.com (H.Z.); yuqf@hunnu.edu.cn (Q.Y.); xiaojun@hunnu.edu.cn (J.X.)

**Keywords:** largemouth bass, proopiomelanocortin (POMC), genotypes, fasting and refeeding, physiological response

## Abstract

Diverse feeding habits in teleosts involve a wide range of appetite-regulating factors. As an appetite-suppressing gene, the polymorphisms of *POMCa* in largemouth bass (*Micropterus salmoides*) were validated via sequencing and high-resolution melting (HRM). The frequency distribution of different *POMCa* genotypes were analyzed in two populations, and physiological responses of different *POMCa* genotypes to feed domestication were investigated. The indel of an 18 bp AU-rich element (ARE) in the 3′ UTR and four interlocked SNP loci in the ORF of 1828 bp of *POMCa* cDNA sequence were identified in largemouth bass and constituted three genotypes of POMC-A I, II, and III, respectively. POMC-A I and Allele I had increased frequencies in the selection population than in the non-selection population (*p* < 0.01), 63.55% vs. 43.33% and 0.7850 vs. 0.6778, respectively. POMC-A I possessed the lowest value of *POMCa* mRNA during fasting (*p* < 0.05) and exhibited growth and physiological advantages under food deprivation and refeeding according to the levels of body mass and four physiological indicators, i.e., cortisol (Cor), growth hormone (GH), insulin-like growth factor-1 (IGF-1), and glucose (Glu). The identification of three *POMCa* genotypes, alongside their varying physiological responses during feed domestication, suggests a selective advantage that could be leveraged in molecular marker-assisted breeding of largemouth bass that are adapted to feeding on formula diet.

## Simple Summary

The domestication of carnivorous largemouth bass by formula diet has been well developed in the aquaculture industry in China. Three distinct *POMCa* genotypes were identified in largemouth bass; significant shifts in genotype frequency were observed between the non-selection population fed on forage fish and the selection population domesticated by formula diet. The random inheritance of allele frequency might be affected by decades of selection on feeding traits of largemouth bass. The increased frequency of POMC-A I in the selection population, along with its association with superior growth and physiological responses, provides novel insights into the genetic adaptation of largemouth bass to artificial diets in aquaculture.

## 1. Introduction

Fish displays diverse feeding behaviors and dietary habits, suggesting the existence of coordination of complex biological processes and endocrine regulation with regard to food intake. Food intake involves a wide range of appetite-regulating factors produced by both the brain and peripheral tissues [1,2], such as orexin [1,3], neuropeptide Y (NPY) [1,3,4], cocaine-amphetamine-regulated transcriptional peptide (CART) [1], and proopiomelanocortin (POMC) [1,5]. These factors affect feeding centers to either stimulate or inhibit feeding.

The hypothalamus contains two main distinct neurons that regulate food intake and energy metabolism: the appetite-promoting agouti-related protein (AgRP) neurons and appetite-suppressing proopiomelanocortin (POMC) neurons [6,7]. POMC neurons in the paraventricular nucleus of the hypothalamus (PVN) release the archetypal polypeptide precursor, which is tissue-specifically cleaved to give rise to a number of functional hormones and neuropeptides, including α-, β-, and γ-melanocyte-stimulating hormone (α-, β-, and γ-MSH), corticotropin-like intermediate lobe peptide (CLIP), adrenocorticotropic hormone (ACTH), endogenous opioid β-endorphin, γ-lipotropin, and met-enkephalin [8,9].

POMC processing generates numerous peptides involved in a variety of functions ranging from stress response to energy homeostasis. These peptides are proteolytically released from POMC by the proprotein convertases [8]. In mammals, POMC neurons are important for the regulation of energy homeostasis and lipid metabolism by regulating the excitability of the sympathetic nervous system [8]. *POMC* mRNA, prohormone convertases (PCs), and β-endorphin have been shown to be involved in the immune processes [10] or relieving neuropathic pain [11] in mammals. ACTH, which could be hydrolyzed to α-MSH and CLIP together, is an important component of the hypothalamic-pituitary-adrenal (HPA) axis in neuroendocrine responses [8]. Much of the focus on the function of α-MSH relates to suppression of food intake through binding to the melanocortin-4 receptors (MC4R) as a primary agonist [5,8,12]. Other melanocortin peptides, such as des-acetyl α-MSH and β-MSH, could be contributing to the similar effect in stimulating cAMP, which is required for activation of MC4R appetite signaling in the PVN [8,13].

In hypothalamic neurons expressing *POMC*, abnormalities in *POMC* processing have a critical impact on the regulation of appetite, energy homeostasis, and body composition [8], and mice and humans carrying deleterious mutations in the *POMC* gene display severe obesity and hyperphagia [14,15,16]. Heterozygosity for a *POMC* mutation had subtle effects on the expression and function of *POMC*, which displayed susceptibility to obesity in a large family of Turkish origin [17]. A 14 bp deletion in the canine *POMC* gene was identified in Labrador retrievers and flat-coat retrievers, and the deletion was associated with greater weight, adiposity, and food motivation in affected dogs. Frequency of the mutation is significantly higher in Labrador retrievers selected to be assistance dogs than pets, suggesting that selection of mutants in the *POMC* signaling pathway may also be involved in the domestication of animals [18].

As in mammals, *POMC* gene signaling pathways are associated with food intake and feeding behavior by releasing satiety-induced melanocortins like α-MSH in teleosts [1,19]. Two copies of the *POMC* gene, A and B, were previously identified in the sturgeon as a result of the teleost genome duplication [20]. Distinct cDNA transcripts of POMC-A1, POMC-A2, POMC-A2s, and POMC-B were discovered in rainbow trout (*Oncorhynchus mykiss*) [21] and Atlantic salmon (*Salmo salar*) [22], presumably attributed to the salmonid-specific genome duplication. However, when investigating the POMC involvement in feeding response, the most primitive copy of POMC-A, defined as POMC-A1 in salmonid fish, was the only transcript that responded significantly to food deprivation [21]. In rainbow trout, fasting was associated with a decrease in hypothalamic expression of *POMC-a1* [21]. Valen et al. [22] suggested feeding in Atlantic salmon induced an increase in *POMC-a1* mRNA abundance in the hypothalamus, while Kalananthan et al. [5] found fasting did not induce any significant changes in the mRNA level of *POMC* paralogs in the hypothalamus between fed and fasted states. Hyperandrogenism had been observed in the *POMCa*-deficient zebrafish (*Danio rerio*), which enhanced somatic growth without increasing adiposity [23]. α-MSH or MC4R agonist inhibited food intake in the goldfish (*Carassius auratus*) [24,25] and rainbow trout [26], suggesting that the MSH/MC4R system plays a crucial role in the anorexigenic regulation of fish.

Largemouth bass (*Micropterus salmoides*) was introduced into China from North America in the 1980s and is now one of the most popular species, with up to 880,000 T/year due to its aquaculture advantages [27]. As a piscivorous fish, commercially cultured largemouth bass used to feed on frozen or live forage fish, which brought about excessive exploitation of fisheries resources and enormous labor costs, and simultaneously, surplus forage fish would deteriorate the water quality sharply. To attenuate the negative effect caused by forage fish, it has become a trend to use formula feed as a substitute in the largemouth bass production.

Largemouth bass have been domesticated by alternative diets for more than ten years [28,29,30]. Varieties or populations feeding on formula diet have been selected and cultured in the majority of fish farms in China now [31,32]. However, some individuals of largemouth bass exhibited poor performance during feed domestication, such as low feed utilization and slow growth. Meanwhile, some fish intolerant of formula feed showed low immunity, high disease susceptibility, and hepatic syndrome such as adipose infiltration and metabolic disorders. All these problems restrict the sustainable development of the industry of largemouth bass. Diverse feeding habits in teleosts involve a wide range of appetite-regulating factors. It would be helpful to focus on the endocrine feeding physiology of candidate genes in largemouth bass feeding on alternative diets.

POMC is known to play an important role in appetite suppression and energy expenditure in both mammals and teleosts, and as a precursor of several bioactive peptides, it releases melanocortin neuropeptides inducing satiety through their interaction with MC4R [1,5]. Although the endocrine mechanisms underlying feeding regulation are conserved among vertebrates, as the most diverse group of vertebrates, only relatively few fish species have been studied regarding the endocrine regulation of food intake. There may be different patterns of feeding regulatory mechanisms specific to diverse fish species. Meanwhile, the role of *POMC* gene mutation has been less recognized in teleosts, and the question arises as to whether the genetic polymorphisms of *POMC* affect the regulation of food intake and appetite suppression in largemouth bass during domestication.

In this study, two populations of largemouth bass, one fed on forage fish consistently (referred to as the “non-selection population” in this study) and the other domesticated by formula diet (referred to as the “selection population”), were collected. *POMCa* was retrieved from the DEGs with SNP annotation in the transcriptome datasets of largemouth bass. Three genotypes with SNPs and insertion/deletion were identified, and the frequency distribution of different genotypes was calculated in the non-selection population and selection population. The expression patterns of *POMCa* were compared in three genotypes after fasting and refeeding treatment. Moreover, various physiological indicators of feeding, including body mass, cortisol (Cor), growth hormone (GH), insulin-like growth factor-1 (IGF-1), and blood glucose (Glu), were measured, and their correlations with *POMC* genotypes were analyzed. This study aims to investigate the endocrine physiology related to gene polymorphisms of *POMCa* in largemouth bass during feed domestication and provide a theoretical basis for the selection and breeding of varieties that are more adapted to feeding on a formula diet. Domestication of largemouth bass by formula diet has been well developed in the aquaculture industry, and this research would benefit the molecular marker-assisted breeding of largemouth bass.

## 2. Materials and Methods

### 2.1. Fish and Sample Collection

Two populations of largemouth bass, a non-selection population and a selection population, were collected from the reservoir in Huarong, Hunan, and fish farms in Foshan, Guangdong, China. Fish were genotyped and implanted with PIT tags. During sample collection, the fish, with an average size of 210.48 ± 47.06 g and 22.28 ± 1.39 cm, were anesthetized with Tricaine (TCI, Tokyo, Japan) at a dosage of ~50 mg/L and then dissected. Peripheral blood was taken from the caudal veins for the detection of physiological indexes including Cor, GH, IGF-1, and Glu. Fish tissues were collected from each individual and preserved in ethanol and RNA*later* (Thermo Fisher Scientific, Waltham, MA, USA) until total DNA and RNA were isolated.

### 2.2. Identification of POMCa and Phylogenetic Analysis

Unigene sequences of *POMC* cDNA with annotation of SNP loci in largemouth bass were obtained from the transcriptomic database, and DNA sequences were retrieved from the genomic database of largemouth bass published in NCBI: https://www.ncbi.nlm.nih.gov/genome/10791?genome_assembly_id=1468587 (accessed on 28 December 2023) [33]. Specific primers aimed at *POMCa* were designed by Primer Premier 5.0 software (Table S1). Total RNA was extracted from 10–30 mg of hypothalamus using Trizol reagent (Invitrogen, Carlsbad, CA, USA). One μg of total RNA was converted into RACE-ready first-strand cDNA, and full-length amplification of *POMCa* cDNA was performed according to the protocol of the SMARTer RACE 5′/3′ Kit (Clontech, Takara, Osaka, Japan). Homologous sequences of other Perciform fishes were obtained from GenBank by BLAST: https://blast.ncbi.nlm.nih.gov/Blast.cgi (accessed on 30 June 2024) (Table S2). Multiple sequence alignments were performed using the Clustal Omega program: https://www.genome.jp/tools/clustalw/; https://www.ebi.ac.uk/Tools/msa/clustalo/ (accessed on 30 June 2024) and DNAMAN v9, and the phylogenetic tree was constructed using the neighbor-joining (NJ) method in MEGA-X software.

### 2.3. Screening and Genotyping of POMCa in Largemouth Bass

The primer pair of POMC-F1/R1 (Table S1) was used for the preliminary screening of mutations in the open reading frame (ORF) and 3′ untranslated region (UTR) of the largemouth bass *POMCa* gene (abbreviated as LMB *POMCa*). The PCR reactions were performed in a final volume of 50 μL according to the protocol of premix Taq (Takara), with the following cycling conditions: (i) 95 °C for 5 min; (ii) 34 cycles of 95 °C for 30 s, 60 °C for 40 s, and 72 °C for 1 min; and (iii) 72 °C for 10 min. PCR products from 10 females and 10 males were sequenced and analyzed by Generay Biotech (Shanghai) Co., Ltd. (China).

An 18 bp indel (insertion/deletion) was found in the 3′ UTR of the *POMCa* gene, and four SNP loci closely linked to the 18 bp indel were screened in the ORF of LMB *POMCa*. Both sequencing and high-resolution melting (HRM) were applied to the genotyping of the *POMCa* gene. POMC-F2/R2 (Table S1) located in the exon 3 of LMB *POMCa* were used for genotyping by sequencing. Pgene-F3/R3 aimed at the second SNP locus were designed for genotyping by HRM. The protocol of HRM detection was as follows: Total DNA was extracted from 10–20 mg of fin tissue using a TIANamp Marine Animal DNA kit (Tiangen Biotech, Beijing, China). DNA quality and concentration were determined using 1.5% agarose gel electrophoresis and a multi-function microplate reader (Biotek Cytation 5, East Lyme, CT, USA), respectively. The PCR reactions were performed in a final volume of 20 μL according to the protocol of the HRM Analysis kit (EvaGreen) (Tiangen Biotech, Beijing, China). The 2-step PCR reaction conditions were: (i) 95 °C for 2 min; (ii) 40 cycles of 95 °C for 10 s and 57 °C for 30 s. Melting curves were generated and analyzed with a CFX96 Real-Time PCR instrument and Precision Melt Analysis v1.2 software (Bio-Rad, Hercules, CA, USA) over a 65–95 °C range.

180 individuals of the non-selection population and 214 individuals of the selection population were chosen to investigate the frequency distribution of *POMC* genotypes. Both genotypes and body weights of all the individuals were recorded. The genetic structure of the top 10%, 20%, and 30% of the largest and smallest individuals were statistically analyzed in two populations.

### 2.4. Fasting and Refeeding Treatment of Fish Fed on Formula Diet

Fish were genotyped, implanted with PIT tags, and then divided into two groups randomly: the control group and the treatment group. Each group consisted of three replicates and stocked with experimental fish at a density of 2.5 kg/m^3^. Fish were kept in indoor cement pools with dimensions of 3 m × 3 m × 1.5 m, continuously supplied with ambient air (dissolved oxygen ≥ 4 mg/L) and maintained at a temperature of 24–26 °C. Fish were fed on a formula diet of commercial pellets (Grobest, Foshan, China; crude protein ≥ 48, crude fat ≥ 6, crude ash ≤ 16, lysine ≥ 3%, calcium ≤ 1~4%). Before the treatment, fish were cultured for one month to adapt to the environment, and the initial weights of fish in different genotypes were recorded. The treatment group was fasted for 3 days (3 d), 1 week (7 d), and then refed for 3 days (10 d) and 1 week (14 d). Considering that largemouth bass took approximately two weeks to recover to their normal level of feed consumption after fasting (1–1.5% of the average body mass), finally, fish continued to be fed for another two weeks before being weighed.

### 2.5. Transcriptional Levels of LMB POMCa During Fasting and Refeeding

At each time of treatment, fish tissues were collected from each genotype in two groups. RNA was extracted from 10–30 mg of tissue using Trizol reagent (Invitrogen, Carlsbad, CA, USA). Next, 1 μg total RNA from each sample was treated with DNase I, and cDNA was synthesized via a PrimeScript RT reagent Kit with gDNA Eraser (Takara, Osaka, Japan). The expression of *POMC* was detected in different tissues, including the pituitary, hypothalamus, head kidney, heart, liver, spleen, kidney, intestine, muscle, and gonad, using the primer pair of POMC-F2/R2 (Table S1).

Afterwards, transcriptional levels of the *POMC* gene in the hypothalamus of mixed genotypes (*n* = 9) and different genotypes (*n* = 3 for each genotype) were investigated using the primer pair of POMC-F3/R3, which was located in the 5′ of LMB *POMCa* covering the α-MSH coding region. qRT-PCR reactions were performed in a final volume of 20 μL using the Power SYBR Green Master Mix in the ABI 7500 Real-Time PCR system (Applied Biosystems, Carlsbad, CA, USA). The PCR reaction conditions were as follows: (i) 95 °C for 120 s; (ii) 40 cycles of 95 °C for 15 s, 60 °C for 15 s, and 72 °C for 30 s. A dissociation curve was generated as follows: 95 °C for 15 s, 60 °C for 60 s, and 95 °C for 15 s. Eukaryotic translation elongation factor 1 alpha 1 (*EF-1α*) and 18S ribosomal RNA (*18S rRNA*) were used as reference genes [32]. The relative expression of the target gene was analyzed using the 2^−ΔΔCT^ method following our previous study [34].

### 2.6. Measurement of Plasma Cor, GH, IGF-1, and Glu

At each time of treatment, blood samples were drawn from at least 9 individuals of each genotype and then centrifuged at 5000 rpm for 15 min to rapidly isolate the plasma. Plasma samples were stored at 2–8 °C and to be analyzed within 24 h. The Cor, GH, and IGF-1 contents were measured using radioisotope (^125^ I)-labeled radioimmunoassays (RIAs), and the Glu content was measured following the glucose oxidase method, as described in previous studies on other Perciform fishes, including Tilapia *(Oreochromis mossambicus*) [35], European sea bass (*Dicentrarchus labrax*) [36], Japanese sea bass (*Lateolabrax japonicus*) [37], and gilt-head seabream (*Sparus aurata*) [38]. Cor, GH, and IGF-1 were measured with Iodine (^125^ I) cortisol, growth hormone, and IGF-1 RIA kits using the XH6080 radioimmunoassay analyzer (National Nuclear Corporation, Beijing, China) respectively. Glu was determined with the Glucose (GO) Assay Kit (Sigma-Aldrich (Shanghai) Trading Co. Ltd., Shanghai, China) using the Toshiba FR120 biochemical analyzer. All the measurements and commercially available kits were provided by the Beijing North Institute of Biotechnology (Beijing, China).

### 2.7. Data Analysis

For the analysis of frequency distribution of *POMC* genotypes, the χ^2^ test was conducted to examine whether the observed genotype frequencies were consistent with the *Hardy-Weinberg* expectations in two populations, and Analysis of Variance (ANOVA) was used to calculate the odd ratio (OR) of different genotypes. For the comparison of levels of LMB *POMCa* and physiological factors in response to fasting and refeeding, all data were presented as mean ± standard error (mean ± SE). Normality was evaluated using the Shapiro-Wilk test before the statistical significance of differences was determined. If the test of normality were violated, the Kruskal–Wallis H test was applied to conduct the non-parametric test. If the test of normality were not violated, all the data were analyzed by a two-way ANOVA using SPSS Statistics 26 (IBM, Armonk, NY, USA), with *p* < 0.05 regarded as significantly different.

## 3. Results

### 3.1. Homology and Structure Analysis of the Appetite-Suppressing Gene, POMC

**Figure 1:**
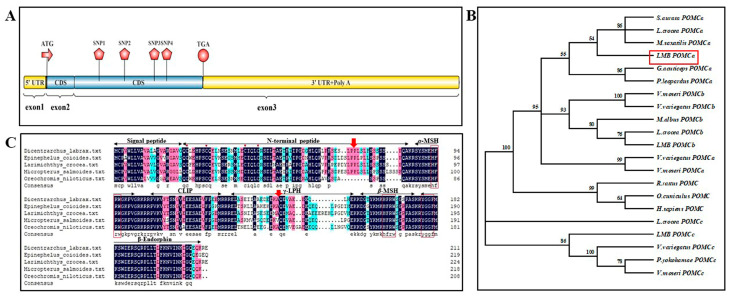
Structure of *POMC* cDNA and phylogenetic analysis of amino acid sequences between largemouth bass and other Perciform fishes. (**A**) 1828 bp of *POMC* cDNA sequence in largemouth bass, including a 96 bp of 5′ UTR, a 1075 bp of 3′ UTR, and a 657 bp of ORF. (**B**) Phylogenetic analysis of *POMC* in Perciformes. (**C**) POMC structure was analyzed in largemouth bass and several other Perciform fishes. The arrows indicated a set of conserved four cysteine residues located at NPP of the molecule. The boxes indicated the core motifs of peptide precursors of the *POMC* gene family: the “YGGF” sequence of β-endorphin and a core sequence of “HFRW” in α-MSH and β-MSH peptides.

The full-length *POMC* cDNA sequence in largemouth bass was 1828 base pairs (bp), including a 96 bp 5′ UTR, a 1075 bp 3′ UTR, and a 657 bp ORF, predicted to encode a protein of 218 amino acids (AAs) and consist of three exons (Figure 1A and Figure S1).

Phylogenetic analysis showed the *POMC* sequence of largemouth bass amplified in this study was closely related to the clusters of proopiomelanocortin-A and could be defined as *POMCa* (Figure 1B). The complete ORF of LMB *POMCa* shared 83.33–86.45% of identity with those of nine Perciform fishes (Table S3). The identity of the deduced AA sequence of LMB *POMCa* with other Perciform fishes ranged from 69.87–80.44%, of which the highest identity was with that of yellow perch (*Perca flavescens*) (80.44%), followed by European sea bass (77.52%), yellow croaker (*Larimichthys crocea*) (77.33%), and giant grouper (*Epinephelus lanceolatus)* (77.27%) (Table S3).

Multiple sequence alignments revealed that LMB *POMCa* contains characteristic conserved domains that could generate a variety of active peptides, including signal peptide, N-terminal region (NPP), α-melanocyte stimulating hormone (α-MSH), CLIP, γ-lipotrophin (γ-LPH), β-MSH, and β-endorphin (β-EP) (Figure 1C). Deduced protein sequence of LMB *POMCa* has the feature sequence of “YGGF” in β-endorphin at the position of 187–190 bp, as shown in Figure 1C. As members of the *POMC* gene family, NPP of the molecule retained a set of four conserved cysteine residues at the positions of 23 bp, 29 bp, 41 bp, and 45 bp, respectively, and both α-MSH and β-MSH could be easily identified by a core sequence of “HFRW” at the positions of 99–102 bp and 175–178 bp (Figure 1C).

### 3.2. Identification of Three Genotypes POMC-A I, POMC-A II, and POMC-A III

**Figure 2:**
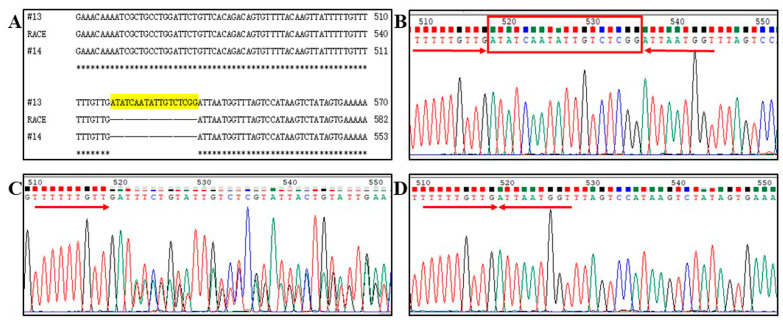
The indel of an 18 bp ARE “ATATCAATATTGTCTCGG” was found in the 3′ UTR of LMB *POMCa*. (**A**) “RACE” represents the sequence of 3′ UTR amplified according to the protocol of the SMARTer RACE 5′/3′ Kit. #13 and #14 represent the sequences of 3′ UTR amplified by the primer pair of POMC-F1/R1. “*” represents identical. (**B**) Chromatogram of POMC-A I 3′ UTR with an 18 bp homozygous insertion. (**C**) Chromatogram of POMC-A II 3′ UTR with a coexistence of the 18 bp ARE insertion and deletion. (**D**) Chromatogram of POMC-A III 3′ UTR with an 18 bp homozygous deletion.

The indel of an 18 bp AU-rich element (ARE) “ATATCAATATTGTCTCGG” was found at 234 bp in the 3′ UTR region of LMB *POMCa*, which constituted three genotypes: POMC-A I with an 18 bp homozygous insertion (Figure 2A,B), POMC-A II with a coexistence of the 18 bp ARE insertion and deletion (Figure 2A,C), and POMC-A III with an 18 bp homozygous deletion (Figure 2A,D).

**Figure 3:**
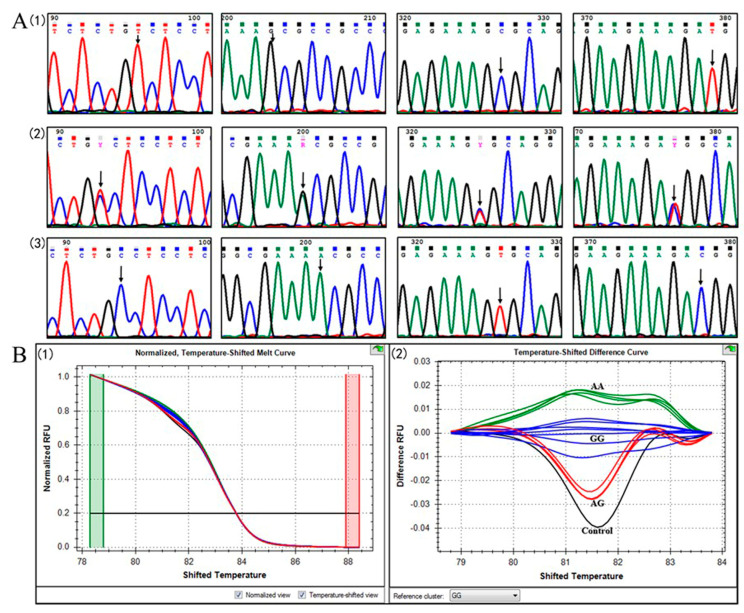
Genotyping of three *POMCa* genotypes by sequencing and HRM detection in largemouth bass. (**A**) SNP Chromatogram of three *POMCa* genotypes. Four SNP loci are tightly linked and indicated by arrows. (1) The homozygote POMC−A I with 220TT/327GG/452CC/504TT; (2) The heterozygote POMC−A II with 220CT/327AG/452CT/504CT; (3) The homozygote POMC−A III with 220CC/327AA/452TT/504CC. (**B**) Genotyping of three *POMCa* genotypes by HRM detection in largemouth bass. (1) Normalized temperature-shifted melting curve. (2) Temperature-shifted difference curve. The blue curve corresponds to the genotype of POMC−A I, the red indicates POMC−A II, and the green profiles represent POMC−A III. The blank control is shown in black, with GG as the reference cluster.

Four SNP loci, located at 220 bp (T220C), 327 bp (G327A), 452 bp (C452T), and 504 bp (T504C) in the exon 3 of LMB *POMCa* ORF, were identified by both sequencing and HRM (Figure 3). The transition of T220C led to the substitution of Serine (S) with Proline (P) at position 74 of the AA sequence (S74P), and C452T resulted in the replacement of Alanine (A) with Valine (V) at position 151 (A151V) (Figure S2). Both SNPs of G327A and T504C were synonymous mutations and did not bring about any amino acid changes (Figure S2). Four SNP loci are tightly linked and, meanwhile, closely interlocked to the 18 bp ARE indel in the 3′ UTR region of LMB *POMCa*. The homozygotes POMC-A I and POMC-A III, and the heterozygote POMC-A II are distinguished by 220TT/327GG/452CC/504TT (Figure 3A-1), 220CC/327AA/452TT/504CC (Figure 3A-2), and 220CT/327AG/452CT/504CT (Figure 3A-3), respectively.

70 bp of amplicon, including the SNP allele of G327A, was subjected to HRM detection for accurate, fast, and massive screening of three genotypes. A temperature shift was applied to normalize all melting curves to the specified intensity threshold. According to the defined ranges of 81.2–81.6 °C, the normalized temperature-shifted melting curve (Figure 3B-1) and difference curve (Figure 3B-2) were displayed in Figure 3B. Three genotypes of POMC-A I (GG), POMC-A II (GA), and POMC-A III (AA) were distinguished based on different melting curve shapes and melting peaks at the temperature of 81.2–81.6 °C (Figure 3B).

### 3.3. Frequency Distribution of Three POMCa Genotypes in Largemouth Bass

**Figure 4:**
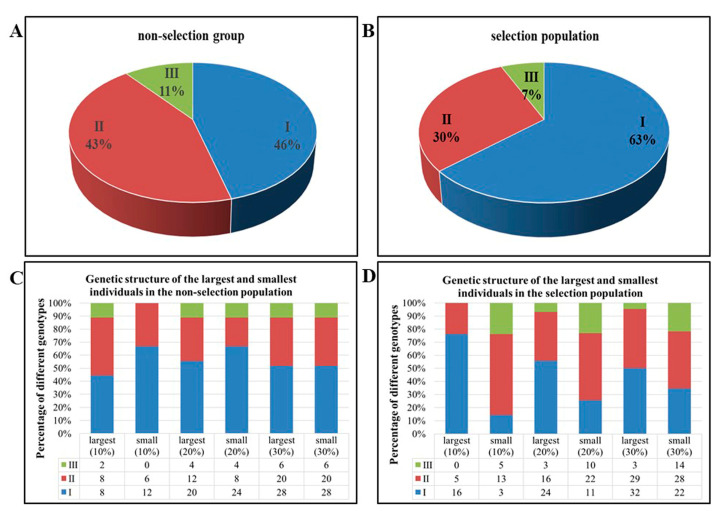
Frequency distribution of three *POMCa* genotypes in largemouth bass. (**A**,**B**) Percentages of three genotypes in non-selection population (**A**) and selection population (**B**), respectively. (**C**,**D**) The genetic structure of the top 10%, 20%, and 30% of the largest and smallest individuals in the non-selection population (**C**) and selection population (**D**), respectively.

Three *POMCa* genotypes were screened and identified in 180 individuals of the non-selection population and 214 individuals of the selection population, respectively (Table 1). In the non-selection population, POMC-A I, POMC-A II, and POMC-A III accounted for 46.11%, 43.33%, and 10.56% of the individuals (Figure 4A), and the frequency of LMB *POMCa* alleles, referred to as Allele I and Allele III, were 0.6778 and 0.3222, respectively (Table 1). While in the selection population, the frequencies of the three genotypes were 63.55%, 29.91%, and 6.54% of the individuals (Figure 4B), and the frequencies of Allele I and Allele III were 0.7850 and 0.2150, respectively (Table 1). According to the law of genetic equilibrium, observed genotype frequencies in both populations were consistent with the *Hardy-Weinberg* expectations (*p* > 0.05) (Table 1). However, frequency distributions between both populations were significantly different (χ^2^ = 12.168, *p* < 0.01) (Table 1). Compared to the genotypes of POMC-A II and POMC-A III, the frequency of POMC-A I in the selection population increased significantly more than that in the non-selection population, with OR (I/II) = 2.003 (*p* < 0.01) and OR (I/III) = 2.230 (*p* < 0.05) (Table 2). There was no significant difference between the frequency distribution of POMC-A II and POMC-A III in the two populations with OR (III/II) = 0.899 (*p* > 0.05) (Table 2). Compared to Allele III, the frequency of Allele I in the selection population increased significantly more than that in the non-selection group with OR (Allele I/Allele III) = 1.739 (*p* < 0.01).

As shown in Figure 4C, in the non-selection population, POMC-A I accounted for 44.44% (*n* = 8) and 55.56% (*n* = 20) of the top 10% (*n* = 18) and 20% (*n* = 36) of the largest individuals, respectively, less than 66.67% (*n* = 12) and 66.67% (*n* = 24) in the top 10% (*n* = 18) and 20% (*n* = 36) of the smallest individuals (*p* < 0.05). POMC-A II accounted for 44.44% (*n* = 8) and 33.33% (*n* = 12) of the top 10% (*n* = 18) and 20% (*n* = 36) of the largest individuals, respectively, greater than 33.33% (*n* = 6) and 22.22% (*n* = 8) in the top 10% (*n* = 18) and 20% (*n* = 36) of the smallest individuals (*p* < 0.05). There were no significant differences between the percentages of POMC-A III in the top 20% (*n* = 36) of the largest and smallest individuals and between the percentages of three genotypes in the top 30% (*n* = 54) of the largest and smallest individuals (*p* > 0.05).

As shown in Figure 4D, in the selection population, POMC-A I accounted for 76.19% (*n* = 16), 55.81% (*n* = 24), and 50.00% (*n* = 32) of the top 10% (*n* = 21), 20% (*n* = 43), and 30% (*n* = 64) of the largest individuals, respectively, which were much greater than 14.29% (*n* = 3), 25.58% (*n* = 11), and 34.38% (*n* = 22) in the top 10% (*n* = 21), 20% (*n* = 43), and 30% (*n* = 64) of the smallest individuals (*p* < 0.01). POMC-A II accounted for 23.81% (*n* = 5) and 37.21% (*n* = 16) of the top 10% (*n* = 21) and 20% (*n* = 43) of the largest individuals, respectively, which were much less than 61.90% (*n* = 13) and 51.16% (*n* = 22) in the top 10% (*n* = 21) and 20% (*n* = 43) of the smallest individuals (*p* < 0.01), and it accounted for 45.31% (*n* = 29) and 43.75% (*n* = 28) in the top 30% (*n* = 64) of the largest and smallest individuals in the selected population with no significant difference (*p* > 0.01). POMC-A III accounted for 0.00% (*n* = 0), 6.98% (*n* = 3), and 4.69% (*n* = 3) of the top 10% (*n* = 21), 20% (*n* = 43), and 30% (*n* = 64) of the largest individuals in the selection population, respectively, which were much smaller than 23.81% (*n* = 5), 23.26% (*n* = 10), and 21.87% (*n* = 14) in the top 10% (*n* = 21), 20% (*n* = 43), and 30% (*n* = 64) of the smallest individuals (*p* < 0.01).

### 3.4. Differential mRNA Expression of LMB POMCa in Response to Fasting and Refeeding

Among the tissues examined, obvious bands of LMB *POMCa* were detectable in the pituitary, hypothalamus, and ovary of the individuals derived from the population domesticated by formula diet; weak bands were observed in the head kidney, heart, liver, and kidney, and no bands were amplified in the spleen, intestine, muscle, and testis (Figure S3).

**Figure 5:**
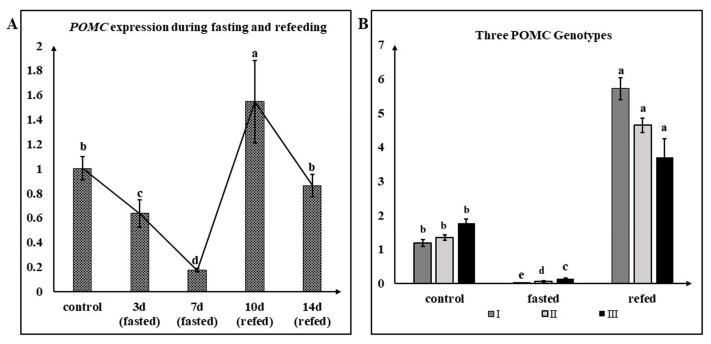
Transcriptional levels of LMB *POMCa* in response to fasting and refeeding. (**A**) *POMC* expression in mixed genotypes (*n* = 9) of largemouth bass fasted for three days (3 d), one week (7 d), and then refed for three days (10 d) and one week (14 d). *n* = 3 for each genotype. (**B**) Differential expression of three *POMCa* genotypes in the treatment group, which was fasted for one week and then refed for three days. Fold changes of three *POMCa* genotypes in different groups were calculated using the 2^−ΔΔCT^ method. Data were expressed as mean ± SE (*n* = 6 for each genotype). ^a,b,c,d^, and, ^e^ represent the significant differences (*p* < 0.05).

LMB *POMCa* expression of mixed genotypes in response to fasting and refeeding were investigated in the hypothalamus. After three and seven days of fasting, the level of LMB *POMCa* mRNA decreased to 63.55 ± 6.37% and 17.39 ± 0.74% of the control value (*p* < 0.05), respectively (Figure 5A, Table S4). However, LMB *POMCa* expression increased up to 154.53 ± 19.37% of the control value after 3 days of refeeding (*p* < 0.05), while it returned to 86.34 ± 5.19% of the initial level with no significant change after seven days of refeeding (*p* > 0.05) (Figure 5A, Table S4).

Furthermore, LMB *POMCa* expression of three different genotypes were examined after being fasted for one week and then refed for three days. LMB *POMCa* expression levels in the control group, which was regularly fed, did not differ significantly among the three genotypes (*p* > 0.05) (Figure 5B, Table S5). After one week of fasting, LMB *POMCa* mRNA was significantly downregulated across all three genotypes. Specifically, LMB *POMCa* expression in POMC-A I was significantly lower than that in POMC-A II, followed by POMC-A III (*p* < 0.05) (Figure 5B, Table S5). After 3 days of refeeding, LMB *POMCa* mRNA was upregulated by more than 1.9-fold to the control levels, while it did not differ significantly among the three genotypes (*p* > 0.05) (Figure 5B, Table S5).

### 3.5. Differential Physiological Responses to Fasting and Refeeding Among Three Genotypes

**Figure 6:**
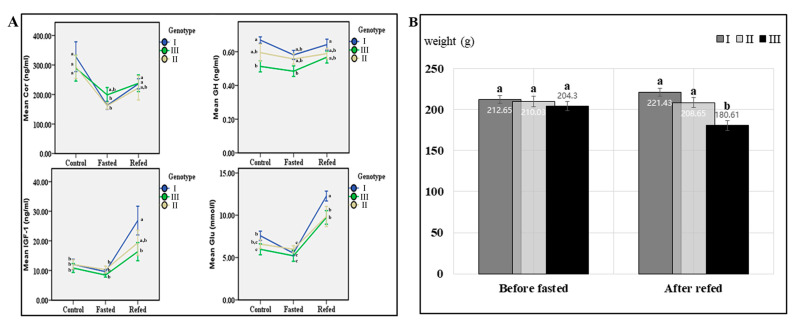
Physiological responses to fasting and refeeding among three *POMCa* genotypes. (**A**) mean values of Cor, GH, IGF-1, and Glu. The blue line corresponds to the genotype of POMC-A I, the yellow indicates POMC-A II, and the green represents POMC-A III. Data were expressed as mean ± SE (n ≥ 9) for each genotype in each group. Thirteen POMC-A I, 9 POMC-A II, and 13 POMC-A III for the control group; 9 POMC-A I, 9 POMC-A II, and 9 POMC-A III for the fasting group; 18 POMC-A I, 9 POMC-A II, and 10 POMC-A III for the refeeding group. (**B**) Body weights (mean ± SE) of three *POMCa* genotypes before fasting and after refeeding. ^a, b,^ and ^c^ represent the significant differences (*p* < 0.05).

Body mass and four physiological indicators (i.e., Cor, GH, IGF-1, and Glu) in response to fasting and refeeding were compared among three genotypes of largemouth bass fed on a formula diet. After one week of fasting, plasma Cor, GH, IGF-1, and Glu levels in all three genotypes decreased as compared with the control group (Figure 6A, Table S6). In contrast, plasma levels of four physiological indicators in three genotypes rose rapidly after 3 days of refeeding (Figure 6A, Table S6). Cor levels in POMC-A I and POMC-A II genotypes and Glu in POMC-A I were sharply down-regulated in response to fasting (*p* < 0.05). Whereas GH and IGF-1 in three genotypes did not change significantly (*p* > 0.05). After refeeding, Cor and GH in all three genotypes recovered to the regular levels (*p* > 0.05), while IGF-1 in POMC-A I and Glu in POMC-A I and POMC-A III increased significantly as compared with the control (*p* < 0.05) (Figure 6A, Table S6). In the control group, GH level in the POMC-A I genotype was significantly higher than that in the POMC-A III genotype (*p* < 0.05). In the refed group, IGF-1 and Glu levels in POMC-A I were also greater than those in the POMC-A III genotype (*p* < 0.05). In addition, there were no significant differences between these indicators of POMC-A I and POMC-A II (*p* > 0.05) (Figure 6A, Table S6).

Three genotypes had average pre-fasting body weights of 212.65 ± 4.80 g (POMC-A I), 210.03 ± 6.36 g (POMC-A II), and 204.3 ± 5.88 g (POMC-A III), respectively, with no significant difference (*p* > 0.05) (Figure 6B, Table S7). After one week of fasting and two weeks of refeeding, the mean body mass (221.43 ± 5.26 g) of POMC-A I increased by 4.13% compared to the pre-fasting value and was 22.60% greater than that (180.61 ± 8.32 g) of POMC-A III (*p* < 0.05), which decreased significantly after fasting and refeeding (*p* < 0.05) (Figure 6B, Table S7). The average body mass (208.65 ± 5.27 g) of POMC-A II was slightly lower after fasting and refeeding but did not differ significantly from the average pre-fasting body mass (*p* > 0.05) (Figure 6B, Table S7).

## 4. Discussion

Fish exhibit different feeding habits, commonly known as selective adaptation to food resources, and the adaptation is essentially genetic-dependent. Many of the genes involved in mammalian neuroendocrine signaling pathways were discovered in fish [22]. The details of feed intake and energy homeostasis in fish are beginning to be elucidated. A previous study suggested that, in Characiformes, different responses of appetite-regulating peptides to fasting were related to both feeding habits and family [39].

The *POMC* gene belongs to the opioid/orphanin gene family, which encodes peptide precursors including either the opioid (YGGF) or the orphanin/nociceptin core sequences (FGGF) [9,40]. POMC holds within its structure numerous biologically active peptides, including the main domains: *α*-MSH/ACTH, C-terminal *β*-lipotropin, *β*-endorphin, etc. [8,9]. LMB *POMCa* shares the core motifs of the members of the *POMC* gene family. It possesses 218 amino acids integrating the “YGGF” sequence of β-endorphin. It also retains a set of four conserved cysteine residues at the N-terminal region of the molecule. MSH peptides are recognized by a core sequence of “HFRW”. A striking feature of the melanocortin end-products is the rigorous conservation of the primary sequence of α-MSH and the first 25 amino acids of ACTH. Multiple melanocortin sequences, such as α-MSH/ACTH and β-MSH, but not γ-MSH, emerged in LMB *POMCa*, which is consistent with the fact that the loss of the γ-MSH sequence occurred during the evolution of POMC in the ray-finned fish [20]. Except for the core motifs, members of the POMC peptide family differ substantially among themselves and between species. The alignment between the deduced protein sequence of LMB *POMCa* and other related species in GenBank showed that the POMC α-MSH, CLIP, β-MSH, and β-EP domains were relatively conserved across the Perciformes, whereas the signal peptide, NPP, and γ-LPH domains were less conserved (Figure 1C).

Polymorphism association analysis of candidate genes was an effective way to improve important economic traits such as growth and reproduction in breeding. A study was conducted to investigate the association between the genotypes of the *IGF-I* gene and growth traits in sea bass. The results revealed *IGF-I* polymorphisms have a significant effect on body weight and total length, which could be useful for the marker-assisted selection (MAS) program of sea bass [41]. Two independent *pomca* mutant 1 lines (M1) with an 8-bp deletion and 8-bp insertion were generated by the TALENs technique, respectively [23]. No obvious differences between M1 zebrafish and wild-type (WT) controls at six days post fertilization (dpf) were observed in terms of food intake. However, food intake of M1 adult zebrafish increased by 52% compared to WT siblings at 5 months post fertilization (mpf) [23]. In previous studies, SNP loci associated with growth traits such as body weight, body height, and full length were identified in largemouth bass [42,43]. However, the biological functions of candidate genes and genotypes in relation to appetite regulation and energy metabolism have not been further validated in the population of largemouth bass fed on an alternative diet.

The polymorphisms of LMB *POMCa* among individuals were validated via RACE, direct sequencing, and HRM detection. The SNPs of T220C, G327A, C452T, and T504C are located at the first, third, second, and third nucleotides of their codons (Figure S2). Transitions of G327A and T504C were non-synonymous mutations, whereas the transitions of T220C and C452T led to changes at position 74 (S74P) and 151 (A151V) of the amino acid sequences, respectively (Figure S2). S74P and A151V were located in the region of NPP and γ-LPH separately. Mutations in the NPP, α-MSH, and β-MSH, but not γ-MSH, have strong associations with obesity in mammals [8,44]. Children who have either homozygous or compound heterozygous mutations in the *POMC* gene provide the strongest evidence for a link between mutations in POMC and obesity. By screening obese populations, two patients were found with different heterozygous mutations in exon 2 of the *POMC* gene, which have been implicated in disruption of POMC sorting to the regulatory secretory pathway [45]. It is speculated that the homozygous and heterozygous mutations in LMB *POMCa* might also be related to energy balance and growth performance.

A frameshift deletion mutation in *POMC* is strongly associated with body weight and appetite in Labrador retriever and flat-coat retriever dogs. The mutation is more common in Labrador retrievers selected to become assistance dogs [20]. The genetic structure of largemouth bass populations adapting to being fed on a formula diet has come to be stable after more than ten years of domestication in China (Table 1). However, the frequency distribution of three genotypes between both populations were significantly different (χ^2^ = 12.168, *p* < 0.01) (Table 1). The frequency of POMC-A I increased significantly more than that in the non-selection population by decades of formula diet domestication (*p* < 0.01) (Table 1 and Table 2, Figure 4). Allele I in the selection population domesticated by formula diet (0.7850) increased significantly compared to that in the non-selection populations fed on forage fish consistently (0.6778) (*p* < 0.01) (Table 1). The genetic structure of POMC-A did not differ significantly between the largest and smallest individuals in the non-selection population (*p* > 0.05) (Figure 4C). In contrast, the proportion of POMC-A I in the largest individuals was much greater than that in the smallest individuals of the selection population, while there was a lesser proportion of POMC-A III in the largest individuals than that in the smallest individuals (*p* < 0.05) (Figure 4D). The selection population of largemouth bass has undergone multiple generations of artificial selection during domestication. The artificial selection pressure on largemouth bass may alter the random inheritance patterns of *POMC*, resulting in the observed imbalanced distribution of the POMC-A I between the two populations.

The physiological process of appetite regulation involves a complex integration of peripheral and central signals by the brain [2]. The *POMC* gene in vertebrates is expressed in multiple organs, including the brain and several other peripheral tissues, in previous reports [6,40]. In mammals and fish, the POMC-derived peptide α-melanocyte stimulating hormone (MSH) is speculated to be involved in appetite suppression through its interaction with melanocortin-4 receptors [6,21]. LMB *POMCa* was strongly expressed in the hypothalamus, pituitary, and ovary but weakly or not expressed in other tissues of the individuals derived from the population domesticated by formula diet (Figure S3). Fasting induced a substantial decrease in the level of the *POMCa* mRNA, of which one week of fasting presented the lowest mean values (*p* < 0.05). In addition, refeeding resulted in a rapid rise of LMB *POMCa* significantly (*p* < 0.05) (Figure 5A, Table S4). The results are consistent with most of the previous studies, in which fasting has been shown to affect the central and peripheral mRNA expressions of appetite-regulating factors in a number of fish species, such as zebra fish and rainbow trout, with usually an increase for appetite stimulators and a decrease for appetite inhibitors in fasted compared to fed fish [1]. Actually, when food is scarce, fish are extremely hungry with good appetites. Therefore, when fish are being fasted, the genes that promote appetite will be up-regulated, and the genes that inhibit appetite will be down-regulated. Nevertheless, when there are abundant supplies of food, fish are less hungry with poor appetite. Therefore, once fish are refed, the genes promoting appetite will be down-regulated, and the genes for suppressing appetite will be up-regulated.

Definitely, *POMC*, as an appetite-suppressing factor, was involved in feeding regulation in the largemouth bass. Furthermore, *POMC* mRNA was significantly downregulated across all three genotypes in largemouth bass after one week of fasting. Specifically, *POMC* expression in homozygote POMC-A I was significantly lower than that in heterozygote POMC-A II, followed by homozygote POMC-A III (*p* < 0.05). Neither in the control nor the refed group were there significant differences among the *POMC* expression levels of the three genotypes (*p* > 0.05) (Figure 5B, Table S5). The expression of this appetite inhibitor decreased during fasting, increasing the appetite of fish. qPCR results indicated that *POMC* expression decreased in starved larval zebrafish [46]. It was shown that *POMC* was a regulatory factor that inhibited feeding in Siberian sturgeon (*Acipenser baerii*) via the peripheral nervous system [47]. POMC-A I possessed the lowest value of *POMC* mRNA during fasting, which indicated that, once feeding was resumed, POMC-A I might feed more actively than the other two genotypes and eventually be more likely to exhibit compensatory growth.

Linkage studies suggested patients with a *POMC* polymorphism located on human chromosome 2 had an increased fat mass and serum leptin levels [48]. *POMC* gene deletion in mice gave an initial association between mutations in *POMC* and decreases in food intake and weight [49]. As in mammals, physiological responses to fasting and refeeding associated with three genotypes were further compared in the following studies (Figure 6A, Table S6). It is speculated that a high level of GH in POMC-A I had an effect on the growth axis and induced the elevation of IGF-1 and Glu in the refed group. Meanwhile, elevation of IGF-1 and Glu levels of POMC-A I in the refed group indicated that the metabolic capacity of POMC-A I was stronger than that of the other two genotypes after fasting and refeeding and thus may more easily accumulate energy to support growth. This also explains the greater increase of body mass observed in the genotype of POMC-A I (221.43 ± 52.09 g) after being refed for two weeks, as compared to the other two genotypes (180.61 ± 8.32 g and 208.65 ± 5.27 g) (Figure 6B, Table S7). Overall, POMC-A I showed increased genetic frequency and superior growth and physiological responses during feed domestication and might be the predominant genotype in the largemouth bass population adapted to feeding on a formula diet.

The mechanism by which *POMC* gene polymorphisms affect the appetite and food intake of largemouth bass is still unclear. The 3′ UTR of eukaryotic genes can regulate their spatial and temporal expression according to environmental stresses, and the negative transcription regulation regions in the 3′ UTR usually form stable RNA secondary structures or contain cis-elements specifically recognized by miRNAs to inhibit translation [50,51]. Cis-acting elements in the 3′ UTR, such as AU-rich elements, are important sites for post-transcriptional regulation of mRNA abundance [51,52]. It has been shown that more than 75% of functional mutations in the 3′ UTR occur at sites rich in AU and U motifs, as well as miRNA binding motifs [52]. Several miRNAs interacting with the 3′ UTR of target genes have been reported to affect traits in fish [53], such as aberrant expression of miR-1388-5p, which was associated with sterility in triploid rainbow trout by repressing the expression of spindlin-1 [54]. An insertion of an 18 bp ARE “ATATCAATATTGTCTCGG” was found in the 3′ UTR of LMB *POMCa*. The POMC-A I genotype expressed the lowest *POMC* mRNA values during fasting in feed domestication (*p* < 0.05), which seems to be related to the post-transcriptional regulation. Possible negative regulatory elements for the regulation of gene expression are located within the AU-rich region of the 3′ UTR of LMB *POMCa*. The insertion of the 18 bp ARE in the 3′ UTR region of LMB *POMCa* affects its own expression and translation levels during feed domestication, which down-regulates the appetite-suppressing pathway and leads to an increase in appetite and weight gain. POMC-A genotypes of largemouth bass may regulate their own mRNA stability through an 18 bp AU-rich element in the 3′ UTR region or may influence the binding of specific miRNAs, ultimately altering the activation level of the POMC-associated appetite regulatory pathway involved in the feed domestication of largemouth bass. Meanwhile, SNPs are useful markers associated with certain economic traits and also provide evidence of the genetic basis of adaptation. The changes of amino acids triggered by SNP loci may alter the protein structure and biological function of the *POMC* gene and finally affect physiological characteristics such as appetite and body mass in the largemouth bass. The mechanisms need to be further investigated.

## 5. Conclusions

Three genotypes of POMC-A I, II, and III are characterized by the indel of an 18 bp ARE in the 3′ UTR and four interlocked SNP loci in the ORF of *POMCa* in largemouth bass, respectively. POMC-A I and Allele I have significantly increased frequencies of inheritance in the selection population (*p* < 0.05). In addition, the proportion of POMC-A I in the largest individuals was much greater than that in the smallest individuals of the selection population, while there was a lesser proportion of POMC-A III in the largest individuals than that in the smallest individuals (*p* < 0.05). The random inheritance of allele frequency might be affected by decades of selection on feeding traits of largemouth bass. POMC-A I exhibited growth advantage and superior physiological responses such as decreased satiety, increased appetite and feeding, and weight gain during feed domestication, and could be the dominant genotype. Genetic polymorphisms of *POMCa* were associated with appetite and food intake in largemouth bass domesticated by alternative diets.

## Figures and Tables

**Table 1:** Frequency and distribution of three *POMCa* genotypes in largemouth bass.

Group	Frequency	Count	Genotype *			Allele		c^2^	*p*
			I	II	III	Allele I	Allele III		
non-selection	observed count	180	83	78	19	244/360	116/360	0.018	*p* = 0.991 > 0.05
	observed frequency		46.11%	43.33%	10.56%	0.6778	0.3222		
	expected count	180	82.69	78.62	18.69				
	expected frequency		45.94%	43.68%	10.38%				
selection	observed count	214	136	64	14	336/428	92/428	2.433	*p* = 0.296 > 0.05
	observed frequency		63.55%	29.91%	6.54%	0.7850	0.2150		
	expected count	214	131.87	72.24	9.89				
	expected frequency		61.62%	33.76%	4.62%				
Between Group	-	-	-	-	-	-	-	12.168	*p* = 0.002 < 0.01

* Genotypes of POMC-A I, POMC-A II, and POMC-A III were abbreviated as I, II, and III, while alleles of LMB POMCa were referred to as Allele I and Allele III, respectively.

**Table 2:** Odds ratio (OR) of three *POMCa* genotypes and confidence interval (95%) in selection and non-selection groups.

OR *	95% CI	*p*
selection/non-selection (I/II) = 2.003	1.303–3.081	0.002
selection/non-selection (I/III) = 2.230	1.055–4.712	0.036
selection/non-selection (III/II) = 0.899	0.414–1.949	0.787
selection/non-selection (Allele I/Allele III) = 1.739	1.260–2.400	0.001

* Genotypes of POMC-A I, POMC-A II, and POMC-A III were abbreviated as I, II and III, while alleles of LMB POMCa were referred to as Allele I and Allele III, respectively.

## Data Availability

Data is contained within the article or Supplementary Materials. The original contributions presented in the study are included in the article or Supplementary Materials, and further inquiries can be directed to the corresponding author.

## Supplementary Materials

The following supporting information can be downloaded at https://www.mdpi.com/article/10.3390/ani14243638/s1. Figure S1: POMCa cDNA sequence of largemouth bass (LMB). The sequence in the gray or yellow shadow is the complete ORF of LMB POMCa, and gray or yellow represents different exons. The start codon (ATG), stop codon (TGA), and SNP loci are marked in red font. Figure S2: Polymorphisms of POMC amino acid sequences in largemouth bass; Figure S3: Tissue distribution of LMB POMCa in various tissues of largemouth bass, including head kidney, pituitary, hypothalamus, heart, liver, spleen, kidney, gut, muscle, testis, and ovary; Table S1: Primers used for amplification, genotyping, and mRNA expression of LMB POMCa sequences; Table S2: Homologous sequences of Perciform fishes used for alignment analysis; Table S3: Identity (%) of LMB POMCa with nine other Perciform fishes; Table S4: LMB POMCa fold change of mixed genotypes during fasting and refeeding; Table S5: LMB POMCa fold change of three genotypes during fasting and refeeding; Table S6: Physiological responses of three POMC genotypes in control, fasted, and refed groups; Table S7: Comparison of body weights (mean ± SE) among three POMC genotypes before fasting and after refeeding.

## References

[1] Volkoff H. (2016). The Neuroendocrine Regulation of Food Intake in Fish: A Review of Current Knowledge. Front. Neurosci..

[2] Martins N., Castro C., Oliva-Teles A., Peres H. (2022). The Interplay between Central and Peripheral Systems in Feed Intake Regulation in European Seabass (*Dicentrarchus labrax*) Juveniles. Animals.

[3] Hoskins L.J., Volkoff H. (2012). The comparative endocrinology of feeding in fish: Insights and challenges. Gen. Comp. Endocrinol..

[4] Narnaware Y.K., Peyon P.P., Lin X., Peter R.E. (2000). Regulation of food intake by neuropeptide Y in goldfish. Am. J. Physiol. Regul. Integr. Comp. Physiol..

[5] Kalananthan T., Lai F., Gomes A.S., Murashita K., Handeland S., Rønnestad I. (2020). The Melanocortin System in Atlantic Salmon (*Salmo salar* L.) and Its Role in Appetite Control. Front. Neuroanat..

[6] Zhan C. (2018). POMC Neurons: Feeding, Energy Metabolism, and Beyond. Adv. Exp. Med. Biol..

[7] Sohn J.W. (2015). Network of hypothalamic neurons that control appetite. BMB Rep..

[8] Harno E., Gali Ramamoorthy T., Coll A.P., White A. (2018). POMC: The Physiological Power of Hormone Processing. Physiol. Rev..

[9] Dores R.M., Baron A.J. (2011). Evolution of POMC: Origin, phylogeny, posttranslational processing, and the melanocortins. Ann. N. Y. Acad. Sci..

[10] Maddila S.C., Busch-Dienstfertig M., Stein C. (2017). B Lymphocytes Express *Pomc* mRNA, Processing Enzymes and β-Endorphin in Painful Inflammation. J. Neuroimmune. Pharmacol..

[11] Ishikawa T., Miyagi M., Yamashita M., Kamoda H., Eguchi Y., Arai G., Suzuki M., Sakuma Y., Oikawa Y., Orita S. (2013). In-vivo transfection of the proopiomelanocortin gene, precursor of endogenous endorphin, by use of radial shock waves alleviates neuropathic pain. J. Orthop. Sci..

[12] Israeli H., Degtjarik O., Fierro F., Chunilal V., Gill A.K., Roth N.J., Botta J., Prabahar V., Peleg Y., Chan L.F. (2021). Structure reveals the activation mechanism of the MC4 receptor to initiate satiation signaling. Science.

[13] Pritchard L.E., Armstrong D., Davies N., Oliver R.L., Schmitz C.A., Brennand J.C., Wilkinson G.F., White A. (2004). Agouti-related protein (83–132) is a competitive antagonist at the human melanocortin-4 receptor: No evidence for differential interactions with pro-opiomelanocortin-derived ligands. J. Endocrinol..

[14] Coll A.P., Challis B.G., López M., Piper S., Yeo G.S., O’Rahilly S. (2005). Proopiomelanocortin-deficient mice are hypersensitive to the adverse metabolic effects of glucocorticoids. Diabetes.

[15] Nasif S., de Souza F.S., González L.E., Yamashita M., Orquera D.P., Low M.J., Rubinstein M. (2015). Islet 1 specifies the identity of hypothalamic melanocortin neurons and is critical for normal food intake and adiposity in adulthood. Proc. Natl. Acad. Sci. USA.

[16] Warden C.H., Fisler J.S., Coulston A.M., Boushey C.J., Ferruzzi M.G., Delahanty L.M. (2017). Chapter 21—Genetics of Nonsyndromic Human Obesity, with Suggestions for New Studies from Work in Mouse Models. Nutrition in the Prevention and Treatment of Disease.

[17] Farooqi I.S., Drop S., Clements A., Keogh J.M., Biernacka J., Lowenbein S., Challis B.G., O’Rahilly S. (2006). Heterozygosity for a POMC-null mutation and increased obesity risk in humans. Diabetes.

[18] Raffan E., Dennis R.J., O’Donovan C.J., Becker J.M., Scott R.A., Smith S.P., Withers D.J., Wood C.J., Conci E., Clements D.N. (2016). A Deletion in the Canine POMC Gene Is Associated with Weight and Appetite in Obesity-Prone Labrador Retriever Dogs. Cell Metab..

[19] Shahjahan M., Kitahashi T., Parhar I.S. (2014). Central pathways integrating metabolism and reproduction in teleosts. Front. Endocrinol..

[20] Danielson P.B., Alrubaian J., Muller M., Redding J.M., Dores R.M. (1999). Duplication of the POMC gene in the paddlefish (*Polyodon spathula*): Analysis of gamma-MSH, ACTH, and beta-endorphin regions of ray-finned fish POMC. Gen. Comp. Endocrinol..

[21] Leder E.H., Silverstein J.T. (2006). The pro-opiomelanocortin genes in rainbow trout (*Oncorhynchus mykiss*): Duplications, splice variants, and differential expression. J. Endocrinol..

[22] Valen R., Jordal A.E.O., Murashita K., Rønnestad I. (2011). Postprandial effects on appetite-related neuropeptide expression in the brain of Atlantic salmon, Salmo salar. Gen. Comp. Endocrinol..

[23] Shi C., Lu Y., Zhai G., Huang J., Shang G., Lou Q., Li D., Jin X., He J., Du Z. (2020). Hyperandrogenism in POMCa-deficient zebrafish enhances somatic growth without increasing adiposity. J. Mol. Cell. Biol..

[24] Cerdá-Reverter J.M., Ringholm A., Schiöth H.B., Peter R.E. (2003). Molecular cloning, pharmacological characterization, and brain mapping of the melanocortin 4 receptor in the goldfish: Involvement in the control of food intake. Endocrinology.

[25] Cerdá-Reverter J.M., Schiöth H.B., Peter R.E. (2003). The central melanocortin system regulates food intake in goldfish. Regul. Pept..

[26] Schjolden J., Schiöth H.B., Larhammar D., Winberg S., Larson E.T. (2009). Melanocortin peptides affect the motivation to feed in rainbow trout (*Oncorhynchus mykiss*). Gen. Comp. Endocrinol..

[27] (2024). China Fishery Statistics Yearbook.

[28] Portz L., Cyrino J.E. (2004). Digestibility of nutrients and amino acids of different protein sources in practical diets by largemouth bass *Micropterus salmoides* (Lacepéde, 1802). Aquac. Res..

[29] Tidwell J.H., Coyle S.D., Bright L.A., Yasharian D. (2005). Evaluation of plant and animal source proteins for replacement of fish meal in practical diets for the largemouth bass *Micropterus salmoides*. J. World Aquacult. Soc..

[30] Ren X., Wang Y., Chen J., Wu Y., Huang D., Jiang D., Li P. (2018). Replacement of Fishmeal with a Blend of Poultry Byproduct Meal and Soybean Meal in Diets for Largemouth Bass, *Micropterus salmoides*. J. World Aquacult. Soc..

[31] Bai J.J., Li S.J., Bai J.J., Li S.J. (2019). Chapter 3—Breeding New Varieties of Largemouth Bass. Genetic Breeding and Molecular Marker-Assisted Selective Breeding of Largemouth Bass.

[32] Ma D.M., Fan J.J., Tian Y.Y., Jiang P., Wang J.J., Zhu H.P., Bai J.J. (2019). Selection of reference genes for quantitative real-time PCR normalisation in largemouth bass *Micropterus salmoides* fed on alternative diets. J. Fish. Biol..

[33] Sun C., Li J., Dong J., Niu Y., Hu J., Lian J., Li W., Li J., Tian Y., Shi Q. (2021). Chromosome-level genome assembly for the largemouth bass *Micropterus salmoides* provides insights into adaptation to fresh and brackish water. Mol. Ecol. Resour..

[34] Yan N., Hu J., Li J., Dong J., Sun C., Ye X. (2019). Genomic organization and sexually dimorphic expression of the *Dmrt1* gene in largemouth bass (*Micropterus salmoides*). Comp. Biochem. Physiol. B Biochem. Mol. Biol..

[35] Kajimura S., Hirano T., Visitacion N., Moriyama S., Aida K., Grau E.G. (2003). Dual mode of cortisol action on GH/IGF-I/IGF binding proteins in the tilapia, Oreochromis mossambicus. J. Endocrinol..

[36] Varsamos S., Flik G., Pepin J.F., Bonga S.E., Breuil G. (2006). Husbandry stress during early life stages affects the stress response and health status of juvenile sea bass, Dicentrarchus labrax. Fish. Shellfish. Immunol..

[37] Qian K., Wen H.S., Chi M., Ni M.L., Zhang D.Q., Ding Y.X. (2014). Effects of Exogenous Hormone Injection on the Serum IGF-1 and the Expression of IGF-1 mRNA and IGFBP-1 mRNA in the Liver of Japanese Sea Bass (*Lateolabrax japonicus*). Prog. Fish. Sci..

[38] Montero D., Izquierdo M.S., Tort L., Robaina L., Vergara J.M. (1999). High stocking density produces crowding stress altering some physiological and biochemical parameters in gilthead seabream, *Sparus aurata*, juveniles. Fish. Physiol. Biochem..

[39] Butt Z.D., O’Brien E., Volkoff H. (2019). Effects of fasting on the gene expression of appetite regulators in three Characiformes with different feeding habits (*Gymnocorymbus ternetzi*, *Metynnis argenteus* and *Exodon paradoxus*). Comp. Biochem. Physiol. A Mol. Integr. Physiol..

[40] Navarro S., Soletto L., Puchol S., Rotllant J., Soengas J.L., Cerdá-Reverter J.M. (2016). 60 YEARS OF POMC: POMC: An evolutionary perspective. J. Mol. Endocrinol..

[41] Özcan Gökçek E., Işık R. (2020). Associations between genetic variants of the insulin-like growth factor I (IGF-I) gene and growth traits in European sea bass (*Dicentrarchus labrax* L.). Fish. Physiol. Biochem..

[42] Quan Y.C., Ma D.M., Bai J.J., Liu H., Li S.J., Liu H.Y. (2016). SNPs identification in RNA-seq data of largemouth bass (*Micropterus salmoides*) fed on formulated feed and association analysis with growth trait. Acta Hydrobiol. Sin..

[43] Ma D.M., Quan Y.C., Fan J.J., Hu J., Bai J.J., Liu H. (2018). Development of SNPs related to bait domestication based on largemouth bass (*Micropterus salmoides*) transcriptome and association analysis with growth traits. J. Fish. China.

[44] Lee Y.S., Challis B.G., Thompson D.A., Yeo G.S., Keogh J.M., Madonna M.E., Wraight V., Sims M., Vatin V., Meyre D. (2006). A POMC variant implicates beta-melanocyte-stimulating hormone in the control of human energy balance. Cell. Metab..

[45] Creemers J.W., Lee Y.S., Oliver R.L., Bahceci M., Tuzcu A., Gokalp D., Keogh J., Herber S., White A., O’Rahilly S. (2008). Mutations in the amino-terminal region of proopiomelanocortin (POMC) in patients with early-onset obesity impair POMC sorting to the regulated secretory pathway. J. Clin. Endocrinol. Metab..

[46] Liu S.S., Zhang C.Z., Peng G. (2016). Effects of starvation on the expression of feeding related neuropeptides in the larval zebrafish hypothalamus. Hereditas.

[47] Yuan D., Gao Y., Zhang X., Wang B., Chen H., Wu Y., Chen D., Wang Z., Li Z. (2019). NPY and NPY receptors in the central control of feeding and interactions with CART and MC4R in Siberian sturgeon. Gen. Comp. Endocrinol..

[48] Comuzzie A.G., Hixson J.E., Almasy L., Mitchell B.D., Mahaney M.C., Dyer T.D., Stern M.P., MacCluer J.W., Blangero J. (1997). A major quantitative trait locus determining serum leptin levels and fat mass is located on human chromosome 2. Nat. Genet..

[49] Coll A.P., Fassnacht M., Klammer S., Hahner S., Schulte D.M., Piper S., Tung Y.C., Challis B.G., Weinstein Y., Allolio B. (2006). Peripheral administration of the N-terminal pro-opiomelanocortin fragment 1-28 to Pomc-/- mice reduces food intake and weight but does not affect adrenal growth or corticosterone production. J. Endocrinol..

[50] Miranda K.C., Huynh T., Tay Y., Ang Y.S., Tam W.L., Thomson A.M., Lim B., Rigoutsos I. (2006). A pattern-based method for the identification of MicroRNA binding sites and their corresponding heteroduplexes. Cell.

[51] Griesemer D., Xue J.R., Reilly S.K., Ulirsch J.C., Kukreja K., Davis J.R., Kanai M., Yang D.K., Butts J.C., Guney M.H. (2021). Genome-wide functional screen of 3’UTR variants uncovers causal variants for human disease and evolution. Cell.

[52] Chan J.J., Zhang B., Chew X.H., Salhi A., Kwok Z.H., Lim C.Y., Desi N., Subramaniam N., Siemens A., Kinanti T. (2022). Pan-cancer pervasive upregulation of 3’ UTR splicing drives tumourigenesis. Nat. Cell. Biol..

[53] Bizuayehu T.T., Babiak I. (2014). MicroRNA in teleost fish. Genome. Biol. Evol..

[54] Wang F., Guo F., Ma W. (2020). Abnormal expression of miR-1388-5p and its target spindlin-1 in female triploid rainbow trout (*Oncorhynchus mykiss*). Aquacult. Rep..

