# Supplementary material for: Identification of Three POMCa Genotypes in Largemouth Bass (Micropterus salmoides) and Their Differential Physiological Responses to Feed Domestication
Source: Animals (Basel). 2024 Dec 17;14(24):3638. doi: 10.3390/ani14243638 (PMC11672714; doi:10.3390/ani14243638)
Supplement: Supplementary file 1 [file animals-14-03638-s001.zip › Figure S1 POMCa cDNA Sequence of LMB.pdf]

>LMB POMC

Exon 1

1 gtg gAC GAG AAC AAC AGA AAT CTC TGT CTA ACG GCC AAA CGA CAA 45

46 GAA AAC AAC AGA AAA GAA AGA GAG AGA GAG AGA GAC AGG ACA ACG 90

Exon 2

91 TGA GGA ATG TGT CCT GTG TGG CTA TTG GTG GCT GTG GCG TTG GTG 135
Met Cys Pro Val Trp Leu Leu Val Ala Val Ala Leu Val

136 GGC GTG GCC AGA GGA GGC CTC CTT CAG TGC TTG GAC CAT CCG AGC 180
15 Gly Val Ala Arg Gly Gly Leu Leu Gln Cys Leu Asp His Pro Ser 29

Exon 3

181 TGT CAG GAG GTC AAC TCT GAG AGC AGC ATG ATG GAC TGT ATC CAG 225
30 Cys Gln Glu Val Asn Ser Glu Ser Ser Met Met Asp Cys Ile Gln 44

226 CTC TGT CAC TCT GAC CTC ACT GCT GAG ATA CCC ATC ATC CCA GGC 270
45 Leu Cys His Ser Asp Leu Thr Ala Glu Ile Pro Ile Ile Pro Gly 59

271 GAT GCC CAC CTC CAA CCT CCT CCT CCA TCA GAT CCC GAG TCT CTG 315
60 Asp Ala His Leu Gln Pro Pro Pro Pro Ser Asp Pro Glu Ser Leu 74

316 CCT CCT CTC TCT CTT TTA TCT CCC TCC TCT TCC TTA TCC TCC TCT 360
75 Pro Pro Leu Ser Leu Leu Ser Pro Ser Ser Ser Leu Ser Ser Ser 89

361 CCT CAG GCC AAG CGC TCC TAC TCC ATG GAG CAT TTC CGC TGG GGG 405
90 Pro Gln Ala Lys Arg Ser Tyr Ser Met Glu His Phe Arg Trp Gly 104

406 AAG CCT GTT GGG CGA AAA CGC CGC CCG GTC AAA GTC TAC AGC TCT 450
105 Lys Pro Val Gly Arg Lys Arg Arg Pro Val Lys Val Tyr Ser Ser 119

451 AAC GAC GTG GAG GAG GAA TCA GCC GAG GTT TTC CCT GGA GAG ATG 495
120 Asn Asp Val Glu Glu Glu Ser Ala Glu Val Phe Pro Gly Glu Met 134

496 AGG AGA CGG GAG CTG GCA AGC AAG ATG GTA GCA TCA CAG GAT GAG 540
135 Arg Arg Arg Glu Leu Ala Ser Lys Met Val Ala Ser Gln Asp Glu 149

541 GAG AAA GTG CAG GAG GTG GCC GAA GAG GAG CAG GAG CAG CTC CCG 585
150 Glu Lys Val Gln Glu Val Ala Glu Glu Glu Gln Glu Gln Leu Pro 164

586 GGA GAG AAG AAA GAC GGC ACG TAC AAG ATG AAG CAC TTC CGC TGG 630
165 Gly Glu Lys Lys Asp Gly Thr Tyr Lys Met Lys His Phe Arg Trp 179

631 AGT GGC CCG CCG GCC AGC AAA CGC TAT GGC GGC TTC ATG AAG AGC 675
180 Ser Gly Pro Pro Ala Ser Lys Arg Tyr Gly Gly Phe Met Lys Ser 194

676 TGG GAC GAG CGG AGC CAG AGG CCG CTG CTC ACG TTC TTC AAA AAT 720
195 Trp Asp Glu Arg Ser Gln Arg Pro Leu Leu Thr Phe Phe Lys Asn 209

721 GTC ATC AAC AAA GAC GGA CAG CAG CAG AAG TGA GTG AGG GAG GTG 765
210 Val Ile Asn Lys Asp Gly Gln Gln Gln Lys End 220

766 AAA GGA GAG GGA AGA GAC AGA CAG AAG ATG AGC AGA CAA AGA GAT 810

811 GCC TCC AAA CTA AAT ACA AAA TAT GAT GTT TGT CAT ACT TTC CAA 855

856 AAG CCT GTC TCC TAG AAA TTA AGT TTA TTC ACT GCT AAT TAG TTT 900

901 TTC CAT TTG TGT TCT GAT GAG AAA CAA AAT CGC TGC CTG GAT TCT 945

946 GTT CAC AGA CAG TGT TTT ACA AGT TAT TTT TGT TTT TTG TTG ATT 990

991 AAT GGT TTA GTC CAT AAG TCT ATA GTG AAA AAT GTT CAT CCC ATT 1035

1036 TTT CCA GAG TTG ATG TCG TGT TCT GTC CAA CCA CCA GTC TCT TAC 1080

1081 CAA AAG ATA CCC AGT TTG CAA TGA AAT AAA AAA AAA GAA AAG AAA 1125

1126 AGC AGT AAA TAT TCA CAA CTG TGA CTC TAT ATG AAT TGT TTT TGG 1170

1171 CAT TTT TTG CTT GGA AAA AGA TTT AAA CGA TTA ATT GAT GAT CAA 1215

1216 AAT AGG TGC CTA GTT GTT TCA GCT GAA CAG TTT TTT TGC AAG TGT 1260

1261 AGG AAG GGG CCA CAT TTC TGG ACG GCA CAG GCA GGT GAT TAG GGT 1305

1306 GGA TGG TTG TTG TAC AAA TCT GGG TTC ACA TCC TGA ATG AAA CAT 1350

1351 TTA AAA GGT TAG GGA AAG ATT GTA TTT TGG GGT AAG TGT TAA TAA 1395

1396 ATT AAA AAT GTC TTG TCT CAC TAT TAA GGT TTT TAG TAT AAA ACC 1440

1441 AAG ACC ATG ACA GTT CCT CAC TCT TAA AGG ATC AGC TTC TTA CTA 1485

1486 TGA GTG AAG GAT ACA GTA GTT AAT TAG TAA AGT TTC CCC CAT TAT 1530

1531 GGT GAC ATA CAA CCT AAT CCG GGC TTC ATT AGG TCT CTT TCA AAA 1575

1576 TGT ATT TGC TGT TGT AAA TAA GTA ATA TAT GGT ATA AAT TTA ATT 1620

1621 TCT AGC AGA CAG GGT TAC AGT AGG GTG ACA TTA TCA GTG TCT TCT 1665

1666 GTT TGA CTC ATA TGG GTG TTT AAA AGA TCA AGA TTA AAA GAG AGA 1710

1711 GAA GAA GAT AAT GAT GGG AGA AAA ATG TTT TAT AAA TTG ATT CAT 1755

1756 GTA GAT AAT TGT AAA TGA CAT AAA AAC ATA AAG TTA TTT GCA AGT 1800

1801 AAA AAA AAA AAA AAA AAA AAA AAA AAA A 1828
