# Supplementary material for: Identification of Three POMCa Genotypes in Largemouth Bass (Micropterus salmoides) and Their Differential Physiological Responses to Feed Domestication
Source: Animals (Basel). 2024 Dec 17;14(24):3638. doi: 10.3390/ani14243638 (PMC11672714; doi:10.3390/ani14243638)
Supplement: Supplementary file 1 [file animals-14-03638-s001.zip › Table S1-S7-REVISON.pdf]

Table S1. Primers used for amplification, genotyping and mRNA expression of LMB *POMCa* sequences.

Gene	Primer	Primer Sequence (5′ - 3′)	TM	Amplicon size (bp)	Method
proopiomelanocortin (*POMC*)	PGSP1-3	GCTGCTGTCCGTCTTTGTTGATGA	67.4	745	5′ - RACE PCR
	PGSP2-4	GCCGAGGTTTTCCCTGGAGAGATG	69.5	1357	3′ - RACE PCR
	POMC-F1	ATGTGTCCTGTGTGGCTATTGG	59.8	919/937	ORF amplification
	POMC-R1	CTATAGACTTATGGACTAAACC	60.6		
	POMC-F2	GCTCTGTCACTCTGACCTCA	55.8	539	Genotyping for sequencing/ RT-PCR
	POMC-R2	CCTCCCTCACTCACTTCTGC	57.9		
	Pgene-F3	GAGCATTTCCGCTGGGGG	57.4	70	Genotyping for High Resolution Melting
	Pgene-R3	CGTCGTTAGAGCTGTAGACTTTG	56.6		
	POMC-F3	GAGATACCCATCATCCCAGGCG	62.3	158	qRT-PCR
	POMC-R3	TTCCCCCAGCGGAAATGCT	61.9		
eukaryotic translation elongation factor 1 alpha 1 (*EF-1α*)	RQ697-G4-1F	GTTGCTGCTGGTGTTGGTGAG	61.2	156	Internal control for qRT-PCR (Ma et. al., 2018)
	RQ697-G4-1R	GAAACGCTTCTGGCTGTAAGG	59.5		
18S ribosomal RNA (*18S rRNA*)	L18S-F	CGATGCTCTTAACTGAGTGTC	62.1	96	Internal control for qRT-PCR
	L18S-R	TAGCTGCGGTATTCAGGCGAC	66.0		

Table S2. Homologous sequences of Perciformes fish used for alignment analysis.

Fish Species	GenBank No.	Annotation
Morone saxatilis	XP_035537288.1	proopiomelanocortin a
Plectropomus leopardus	XP_042370656.1	proopiomelanocortin a
Thunnus maccoyii	XP_042260231.1	proopiomelanocortin a
Cheilinus undulatus	XP_041638180.1	proopiomelanocortin a
Gymnodraco acuticeps	XP_034091845.1	proopiomelanocortin a
Rattus rattus	XP_032764475.1	pro-opiomelanocortin
Oryctolagus cuniculus	XP_008253036.1	PREDICTED: proopiomelanocortin
Oreochromis niloticus	QOJ42647.1	proopiomelanocortin b precursor
Larimichthys crocea	QCO31621.1	proopiomelanocortin-C
Larimichthys crocea	QCO31620.1	proopiomelanocortin-B
Larimichthys crocea	QCO31619.1	proopiomelanocortin-A
Oncorhynchus mykiss	NP_001118190.1	pro-opiomelanocortin A precursor
Micropterus salmoides	XP_038587575.1	proopiomelanocortin B
Micropterus salmoides	XP_038582631.1	proopiomelanocortin-C
Micropterus salmoides	XP_038581588.1	proopiomelanocortin-A
Solea senegalensis	CCC15305.1	proopiomelanocortin B
Solea senegalensis	CCA65461.1	proopiomelanocortin a
Homo sapiens	CAG46625.1	POMC, partial
Oncorhynchus mykiss	CAA49467.1	proopiomelanocortin B
Verasper variegatus	BCL66286.1	proopiomelanocortin C
Verasper variegatus	BCL66285.1	proopiomelanocortin B
Verasper variegatus	BCL66284.1	proopiomelanocortin A
Pseudopleuronectes yokohamae	BBO15490.1	proopiomelanocortin-C
Verasper moseri	BAG48197.1	proopiomelanocortin-C
Verasper moseri	BAG48196.1	proopiomelanocortin-B
Verasper moseri	BAG48195.1	proopiomelanocortin-A
Argyrosomus regius	ATZ76924.1	proopiomelanocortin B, partial
Cynoglossus semilaevis	APR72394.1	POMC-B
Cynoglossus semilaevis	AIG92830.1	POMC-a
Dascyllus trimaculatus	AGN30564.1	pro-opiomelanocortin beta, partial
Amatitlania nigrofasciata	AGN30563.1	pro-opiomelanocortin beta
Monopterus albus	AEX97167.1	POMC-B
Monopterus albus	AEX97163.1	POMC-a
Sparus aurata	AEI28996.1	proopiomelanocorticotrophin A
Acipenser transmontanus	AAD55816.1	proopiomelanocortin A precursor
Polyodon spathula	AAD41263.1	proopiomelanocortin A
Acipenser transmontanus	AAD17806.1	proopiomelanocortin B

Table S3. Identity (%) of LMB *POMCa* with nine other Perciformes fish.

Fish species	GenBank No. of mRNA	Length (bp)	Identity(%)	GenBank No. of protein	Length (aa)	Identity (%)	Annotation
Anarrhichthys ocellatus	XM_031878506.1	696	84.65	XP_031734366.1	231	71.23	proopiomelanocortin-like
Cottoperca gobio	XM_029443863.1	657	84.49	XP_029299723.1	218	77.03	proopiomelanocortin-like
Dicentrarchus labrax	AY691808.1	639	85.95	AAU00742.1	212	77.52	proopiomelanocortin
Epinephelus coioides	AY169408.1	660	85.69	AAO11696.1	219	75.91	proopiomelanocortin
Epinephelus lanceolatus	XM_033637918.1	660	86.45	XP_033493809.1	219	77.27	proopiomelanocortin-like
Larimichthys crocea	MH823737.1	678	84.76	XP_010754512.1	225	77.33	proopiomelanocortin-A
Larimichthys crocea	MH823738.1	633	84.16	QCO31620.1	210	76.89	proopiomelanocortin-B
Lates calcarifer	XM_018683013.1	657	84.11	XP_018538529.1	218	76.15	proopiomelanocortin-like
Perca flavescens	XM_028597621.1	705	84.99	XP_028453422.1	234	80.44	proopiomelanocortin-A
Sparus aurata	HM584909.1	690	83.33	AEI28996.1	229	73.06	proopiomelanocorticotrophin A
Sparus aurata	HM584910.1	699	84.78	AEI28997.1	232	69.87	proopiomelanocorticotrophin B

Table S4. LMB *POMCa* fold change of mixed genotypes during fasting and refeeding.

Replicates	1	2	3	Mean	SE
control	1.0000 *	0.9079	1.1015	1.0031 [b]	0.0559
3d (fasted)	0.6291	0.5285	0.7489	0.6355 [c]	0.0637
7d (fasted)	0.1736	0.1612	0.1870	0.1739 [d]	0.0074
10d (refed)	1.5210	1.2226	1.8923	1.5453 [a]	0.1937
14d (refed)	0.8602	0.7751	0.9548	0.8634 [b, c]	0.0519

* represents the reference sample. [a, b, c] and [d] represent the significant difference ($P < 0.05$). Each replicate was consisted of three genotypes.

Table S5. LMB *POMCa* fold change of three genotypes during fasting and refeeding.

Groups	Genotypes	1	2	3	4	5	6	Mean	SE
control	POMC-A Ⅰ	1.0000	1.1096	1.2397	1.2570	1.2226	1.2570	1.1810 [b]	0.1045
	POMC-A II	1.4044	1.4340	1.2746	1.3755	1.2570	1.3195	1.3442 [b]	0.0717
	POMC-A III	1.7654	1.6021	1.6358	1.7411	1.8790	1.9453	1.7615 [b]	0.1336
fasted	POMC-A Ⅰ	0.0157	0.0234	0.0133	0.0219	0.0139	0.0185	0.0178 [e]	0.0042
	POMC-A II	0.0518	0.0600	0.0310	0.0477	0.0647	0.0718	0.0545 [d]	0.0144
	POMC-A III	0.1406	0.1051	0.1015	0.1719	0.1183	0.1571	0.1324 [c]	0.0288
refed	POMC-A Ⅰ	5.6178	5.8563	5.6569	6.2767	5.3889	5.5022	5.7165 [a]	0.3162
	POMC-A II	4.5002	4.2871	4.7240	4.8568	4.6268	4.8232	4.6363 [a]	0.2153
	POMC-A III	3.3404	4.7899	3.4822	3.3870	3.6808	3.4822	3.6937 [a]	0.5496

[*] represents the reference sample. [a, b, c, d] and [e] represent the significant difference ($P < 0.05$).

Table S6. Physiological responses of three *POMC* genotypes in control, fasted and refed groups.

Group	Genotype	Cor (ng/ml)	GH (ng/ml)	IGF-1 (ng/ml)	Glu（mmol/L）
Control	POMC-A I	328.09±50.54 ^{a}	0.67±0.03 ^{a}	12.07±2.12 ^{b}	7.57±0.55 ^{b}
	POMC-A II	293.43±39.66 ^{a}	0.59±0.05 $^{a, b}$	12.04±1.58 ^{b}	6.58±0.53 $^{b, c}$
	POMC-A III	289.20±43.76 ^{a}	0.51±0.03 ^{b}	10.84±1.49 ^{b}	5.97±0.64 ^{c}
Fasted	POMC-A I	163.24±13.76 ^{b}	0.58±0.04 $^{a, b}$	9.61±0.68 ^{b}	5.51±0.28 ^{c}
	POMC-A II	161.87±10.72 ^{b}	0.56±0.05 $^{a, b}$	10.30±1.13 ^{b}	5.97±0.42 ^{c}
	POMC-A III	198.61±25.05 $^{a, b}$	0.48±0.05 ^{b}	8.46±0.67 ^{b}	5.41±0.73 ^{c}
Refed	POMC-A I	236.23±17.19 ^{a}	0.64±0.05 ^{a}	30.77±5.05 ^{a}	12.27±0.59 ^{a}
	POMC-A II	222.54±41.19 $^{a, b}$	0.59±0.05 $^{a, b}$	19.36±4.28 $^{a, b}$	9.85±1.13 ^{b}
	POMC-A III	238.09±28.32 ^{a}	0.57±0.05 $^{a, b}$	16.36±2.90 ^{b}	9.73±0.28 ^{b}

$^{a, b}$ and ^{c} represent the significant difference ($P < 0.05$).

Table S7. Comparison of body weights (Mean±SE) among three *POMC* genotypes before fasting and after refeeding.

Genotype	POMC-A I	POMC-A II	POMC-A III
Before fasted	212.65 ± 4.80 [a]	210.03 ± 6.36 [a]	204.30 ± 5.88 [a]
After refed	221.43 ± 5.26 [a]	208.65 ± 5.27 [a]	180.61 ± 8.32 [b]

[a] and [b] represent the significant difference ($P < 0.05$).
