# Supplementary figures and images for: Identification of Three POMCa Genotypes in Largemouth Bass (Micropterus salmoides) and Their Differential Physiological Responses to Feed Domestication
Source: Animals (Basel). 2024 Dec 17;14(24):3638. doi: 10.3390/ani14243638 (PMC11672714; doi:10.3390/ani14243638)

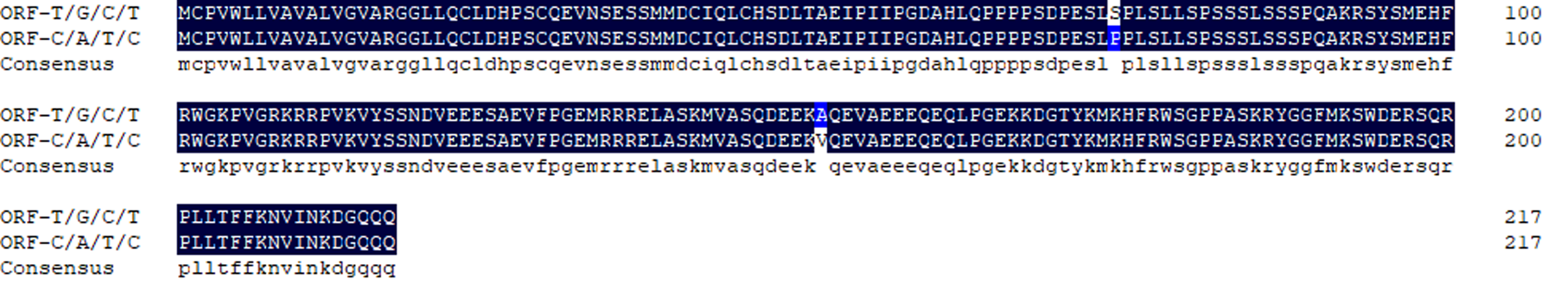

Supplement: Supplementary file 1 [file animals-14-03638-s001.zip › Figure S2 SNP Locus of POMCa in LMB.tif]

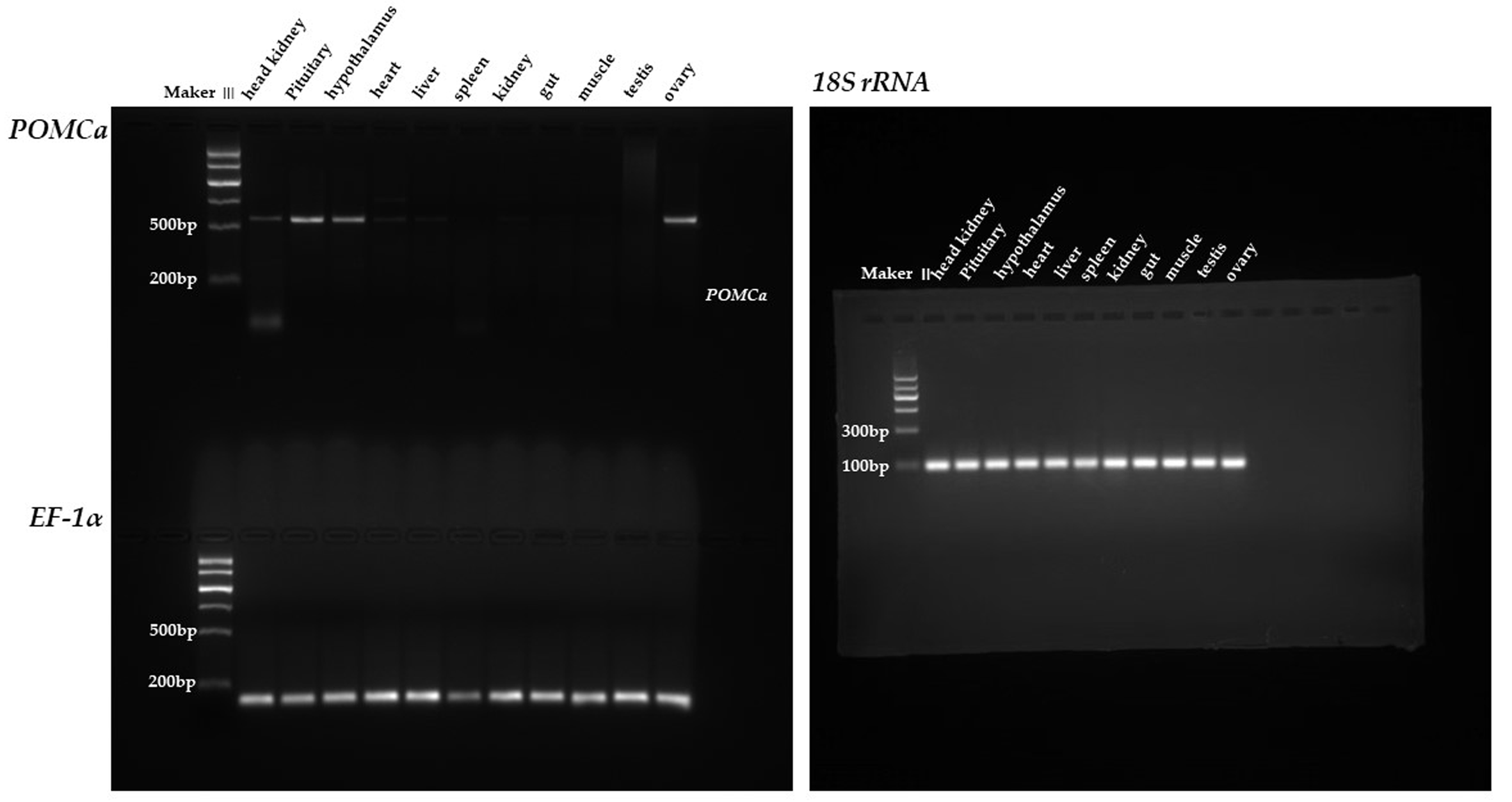

Supplement: Supplementary file 1 [file animals-14-03638-s001.zip › Figure S3 Tissue distribution of LMB POMCa in various tissues-REVISION.tif]
